# Associations Between Perceived Neighbourhood Built Environment and the Maintenance of Regular Walking: A Role of Gender and Socioeconomic Status in a Cross‐Sectional Study

**DOI:** 10.1002/nop2.70360

**Published:** 2025-11-12

**Authors:** Songwhi Noh, Jina Choo, Sooyeon Park, Sae Young Jae

**Affiliations:** ^1^ College of Nursing Korea University Seoul South Korea; ^2^ Transdiciplinary Major in Learning Health Systems, Department of Healthcare Science, Graduate School Korea University Seoul South Korea; ^3^ College of Nursing Konyang University Daejeon South Korea; ^4^ Department of Sport Science University of Seoul Seoul South Korea

**Keywords:** health behaviour, neighbourhood characteristics, socioeconomic factors, urban health, walking

## Abstract

**Background:**

Endorsing the maintenance of regular walking as a form of physical activity is a fundamental component of population‐level health promotion strategies. Among various influencing factors, the neighbourhood‐built environment has been recognised as a key determinant of individuals' walking behaviour. However, evidence on the role of gender and socioeconomic status—both in the prevalence of walking maintenance and in its association with perceived neighbourhood environmental attributes—remains limited and may vary depending on contextual factors, such as metropolitan settings.

**Aim:**

To examine the prevalence of walking maintenance among Seoul citizens and explore the association between perceived neighbourhood environmental attributes and walking maintenance, stratified by gender and socioeconomic status.

**Design:**

A cross‐sectional study design.

**Methods:**

Data were collected from 2000 Seoul residents aged 18–69 years. Walking maintenance was defined as walking ≥ 30 min per day, ≥ 5 days per week, for at least 6 months. Neighbourhood environmental attributes were measured using the Physical Activity Neighbourhood Environment Scale. Analyses were stratified by gender and socioeconomic status.

**Results:**

Women and individuals with lower socioeconomic status were less likely to maintain regular walking compared to their counterparts. Individuals with lower socioeconomic status—but not women—perceived their neighbourhood environmental attributes—particularly aesthetics, recreational facilities, safety and pedestrian/bicycling infrastructure—as less conducive to maintaining regular walking.

**Conclusion:**

Targeted improvements to neighbourhood environments may promote sustained walking, particularly among vulnerable populations, including women and individuals with lower socioeconomic status. These findings support nurse‐led, environment‐focused strategies to enhance long‐term physical activity.

**Implications for the Profession:**

Community health nurses should lead efforts to identify populations at risk of discontinuing regular walking and promote walking maintenance through environmental approaches and collaboration with urban planning initiatives that improve neighbourhood conditions. Such efforts may support sustained walking and contribute to reducing health disparities.

**Patient or Public Contribution:**

No patient or public contribution.

## 1 Introduction

Endorsing regular walking as a form of physical activity constitutes a fundamental component of global population‐level health promotion strategies (Office of the Surgeon General (US) 2015). Walking is the most common and accessible physical activity that is inexpensive or free (Piercy et al. 2018; World Health Organization [WHO] 2024). Engaging in at least approximately 8000 steps of walking per day can reduce all‐cause mortality by 51% (Saint‐Maurice et al. 2020). Despite the benefits of walking, approximately 49.2% of individuals do not achieve the recommended step count needed to attain health benefits (Saint‐Maurice et al. 2020). To effectively promote regular walking among the general population, it is essential first to identify population subgroups with low levels of regular walking.

From this perspective, engagement in regular walking may be influenced by sociodemographic characteristics such as gender and socioeconomic status (SES). Reportedly, women may be a population group more vulnerable than men to overall physical activity participation (Armstrong et al. 2018; The Lancet Public 2019; WHO 2024). However, there is limited empirical evidence on the differences in regular walking between men and women. Individuals with low SES are less likely to participate in regular walking than those with high SES (Kamphuis et al. 2009). Moreover, it was postulated that such a low level of walking observed among socioeconomically disadvantaged populations may be attributed to a variety of interrelated factors. These include individual‐level characteristics such as diminished motivation for physical activity (Bauman et al. 2012), as well as time constraints arising from socioeconomic vulnerability (Bo 2022; Kamphuis et al. 2009). Beyond that, environmental determinants among women and individuals with low SES must also be recognised as critical contributors to this disparity (Ghani et al. 2016; Koohsari et al. 2017). For these populations, promoting walking, typically performed in outdoor settings that require no financial investment or special equipment, can be performed at any time within their own neighbourhoods—may be a practical and impactful strategy. Such an approach holds particular importance for enhancing sustainable physical activity at the population level, especially in resource‐limited contexts.

The World Health Organization has long recognised the critical role of environmental factors in shaping health behaviours and has called on countries to reconfigure supportive environments to promote healthier lifestyles (WHO 2019). Studies also indicated that the neighbourhood‐built environment may influence individuals' walking behaviour (Humpel et al. 2004; Saelens and Handy 2008). Women are more likely to feel physically vulnerable in unsafe environments because of crime‐related safety concerns (Foster and Giles‐Corti 2008). Socioeconomically vulnerable populations were more likely to be affected by crime, conflict, and discrimination and poorly maintained facilities (Stodolska et al. 2011). This suggests that both women and individuals with low SES may be more sensitive than their counterparts to these environmental factors, which play an important role in their participation in regular walking.

Promoting sustainable walking behaviours at the population level necessitates a context‐specific understanding of neighbourhood environmental characteristics across different communities. Seoul is a metropolitan city with a well‐developed public transportation system, pedestrian pathways and transportation infrastructure (Seong et al. 2021). The Seoul Metropolitan City government has implemented several health promotion policies to create walking‐friendly environments, such as expanding urban forest parks, installing walking routes and improving the usability of walking facilities (Lee 2016). A notable example is the Cheonggyecheon restoration project, which has become emblematic of ecological and social urban regeneration (Seoul Solution 2014). These efforts parallel global initiatives, such as New York's High Line, London's Green Grid and Tokyo's Green Biz reflecting a shared commitment to sustainability and improved quality of urban life (Friends of the High Line 2025; Greater London Authority 2014; Tokyo Metropolitan Government 2024). Seoul's strategy illustrates the potential of targeted environmental interventions to foster health and well‐being on a population scale, particularly in high‐density urban environments.

Despite this historical context of urban environmental restructuring, empirical studies on the relationship between environmental factors and citizens' health outcomes remain limited. Therefore, it is necessary to investigate how environmental conditions influence walking behaviours among Seoul residents and to further identify key environmental determinants that enable sustainable walking practices. From a public health perspective, community health nurses increasingly emphasise environment‐centred strategies over individual‐level approaches, especially to promote physical activity among health‐vulnerable populations. Moreover, few studies have indicated that maintenance of regular walking (i.e., long‐term walking), rather than short‐term walking mostly used, would be influenced by people's perceptions of the availability of walkability features as neighbourhood environmental attributes, such as alternative walking routes (Sugiyama, Shibata, et al. 2015).

Therefore, this study examined the prevalence of regular walking maintenance among Seoul citizens, stratified by gender and SES. Furthermore, it explored how perceptions of neighbourhood environmental attributes were associated with the maintenance of regular walking, comparing women and men, as well as individuals with low versus high SES in Seoul, South Korea.

## 2 Methods

### 2.1 Study Design and Study Population

This cross‐sectional, correlational study was conducted through a secondary analysis of data from the Perception of Neighbourhood Environment for a Physical Activity‐Friendly Environment Survey that was a parent survey initiated by the Expert Group for Health Promotion under the Seoul Metropolitan Government, which conducted data collection via an online survey between 22 and 28 September 2020.

The study population consisted of 2000 Seoul residents recruited from a panel maintained by Hyundai Research Co. Ltd., a survey research institute commissioned by the Seoul Metropolitan Government. In 2020, when this study was conducted, the institute had a nationwide panel of approximately 920,000 individuals, of whom about 230,000 were Seoul residents. From this panel, the sample was selected using proportional allocation according to the population structure of Seoul and stratified by gender, age and region. Inclusion criteria comprised individuals aged 18–69 years who resided in Seoul and were registered in the population statistics as of August 2020. The exclusion criterion was the inability to participate in an online survey; specifically, individuals with limited internet access or who were unable to use online platforms were excluded. Panel members registered with Hyundai Research Co. Ltd. received survey invitations via email, text messages and social media platforms (e.g., KakaoTalk). Those who expressed interest in participating accessed the survey by clicking a link provided.

### 2.2 Measures

The sociodemographic characteristics of the study population were collected on age (≥ 40 or < 40), gender (women or men), SES (low or high), obesity status (yes or no), marital status (married or unmarried), education level (≥ college level or < college level) and employment status (employed or unemployed). SES was determined based on the monthly household income (in KRW) using the ‘average monthly decile threshold per household data’ (Statistics Korea 2021). In 2020, the threshold for the 8th decile—representing the top 30% of households by average monthly income—was approximately KRW 6,335,797 (USD 5295). Based on this benchmark, individuals with a monthly household income of less than KRW 6,000,000 were classified as having low SES, while those with an income of KRW 6,000,000 or more were classified as having high SES. Obesity status was determined using body mass index (BMI), calculated as weight in kilograms divided by height in metres squared (kg/m^2^). According to the Asia‐Pacific BMI classification by the WHO (2000), individuals with a BMI of 25.0 kg/m^2^ or more were considered to have obesity, while those with a lower BMI were not considered to have obesity. Furthermore, the study population was categorised into those who owned a personal vehicle (those who owned one or more personal vehicles) and those who did not own a personal vehicle (those who did not own any personal vehicle).

Maintenance of regular walking (i.e., walking^M^) was defined as walking daily for ≥ 30 min five or more days a week and continuing this habit for 6 or more months. It was measured using one question each on the average walking duration per session, the frequency of walking per week and the period of continuance of walking.

Neighbourhood environmental attributes were assessed using the Korean version of the Physical Activity Neighbourhood Environment Scale (PANES) (Sallis 2016; Sallis et al. 2010). The PANES is a 17‐item questionnaire that evaluates physical activity‐related attributes of the environment of one's neighbourhood. The area around one's home where one could walk to in 10–15 min was defined as one's neighbourhood. The PANES includes the attributes of residential density, land use mix, access to transit, pedestrian infrastructure, bicycling infrastructure, recreation facilities, street connectivity, crime safety, traffic safety, pedestrian safety and aesthetics. The original English version of the PANES was translated into Korean by four nursing experts, including the principal investigator. The independently translated version was then harmonised and consolidated through a consensus meeting. The consolidated version was then submitted to a professional translation centre (i.e., Korea University Foreign Language Center, International Writing Services) for back‐translation into English. The back‐translated version underwent further consensus review by the same scholars, resulting in the finalised Korean version used in this study. In 2025, the Korean version of the PANES was psychometrically validated (Kwon 2025), and the validated version demonstrated close comparability with the translated and back‐translated version employed in this study.

In the original survey, all of these attributes, except for residential density, were assessed on a 4‐point Likert scale ranging from ‘strongly disagree’ to ‘strongly agree’. Residential density was determined based on the housing type, which was rated on a 5‐point scale with responses ranging from ‘Detached single‐family housing’ (indicating low residential density) to ‘Apartments or condos of more than 12 stories’ (indicating high residential density). To examine the association with walking^M^, we converted all of these responses to dichotomous variables based on the scale's scoring guidelines—for example, in the case of residential density, 0 indicated ‘single‐family housing’ and 1 indicated ‘all other responses’. The items in the original PANES have shown inter‐rater reliability (kappa = 0.35–0.70) and acceptable validity (rho = 0.31–0.81) (Sallis et al. 2010) and the PANES has been found to be reliable in Sweden and Nigeria (Alexander et al. 2006; Oyeyemi et al. 2008). The attributes of pedestrian infrastructure and bicycling infrastructure were recoded to create an infrastructure variable.

In calculating the PANES total score, all items—except for the personal vehicle ownership item—were coded on a 4‐point scale, with item scores ranging from 1 to 4. Calculated according to the guidelines (Sallis et al. 2010), the total score on the PANES was the average of the scores on the 16 items, and higher scores indicated a more physical activity‐friendly neighbourhood environment (Sallis et al. 2010).

### 2.3 Data Analysis

All statistical analyses were performed using SPSS WIN 28.0 program. We analysed the general characteristics of the study population and the prevalence of walking^M^ according to gender and SES. These data were presented as frequencies and percentages. Chi‐square (*χ* ^2^) tests were conducted to examine differences in general characteristics by gender and SES. Independent *t*‐tests were used to assess differences in the prevalence of walking^M^ and the total score on the PANES based on gender and SES. A *p*‐value of < 0.05 was considered statistically significant.

Perceptions of each neighbourhood environmental attribute were analysed, and the frequencies and percentages of individuals who rated the attribute were reported. Finally, multiple logistic regression analyses were performed—adjusted for age, obesity status, marital status, education level, employment status and ownership of personal vehicle—to identify whether neighbourhood environmental attributes were significantly associated with the walking^M^. Analyses were stratified by gender and SES. All associations were reported as adjusted odds ratios (ORs) with 95% confidence intervals (CIs).

## 3 Results

### 3.1 Sociodemographic Characteristics of the Study Population

Among the total participants (*N* = 2000, aged 18–69 years), 57.6% were aged 40 years or older, 85.9% had attained a college degree or higher, 79.6% were employed and 72.2% owned a personal vehicle (Table 1). The study population comprised 1021 women (51.1%) and 979 men (48.9%), with 71.1% of women and 69.9% of men classified in the low SES group. Among women, 43.0% of those with low SES and 39.7% of those with high SES were aged under 40 years. The prevalence of obesity among women was 11.7%, which was significantly lower than that among men (34.0%; *p* < 0.05). In the low SES group of women, 47.1% were married, 81.8% had attained a college‐level education or higher, 70.0% were employed and 61.2% owned a personal vehicle. These proportions were significantly lower than those observed in the high SES group of women (*p* < 0.05). Among men, 44.6% in the low SES group and 38.6% in the high SES group were under 40 years of age. In the low SES group, 49.3% were married, 84.5% had a college‐level education or higher, 85.2% were employed and 72.8% owned a personal vehicle. These proportions were also significantly lower than those in the high SES group of men (*p* < 0.05).

**TABLE 1:** Sociodemographic characteristics of the study population (*N* = 2000).

Variable	*n* (%)							*p* ^c^
	All	Women (*n* = 1021)			Men (*n* = 979)			
	(*N* = 2000)	Low SES	High SES	*p* ^a^	Low SES	High SES	*p* ^b^	
		(*n* = 726)	(*n* = 295)		(*n* = 684)	(*n* = 295)		
Age (years)								
< 40	848 (42.4)	312 (43.0)	117 (39.7)	0.331	305 (44.6)	114 (38.6)	0.084	0.724
≥ 40	1152 (57.6)	414 (57.0)	178 (60.3)		379 (55.4)	181 (61.4)		
Obesity status								
Yes	452 (22.6)	92 (12.7)	27 (9.2)	0.112	232 (33.9)	101 (34.2)	0.923	< 0.001^c^
No	1548 (77.4)	634 (87.3)	268 (90.8)		452 (66.1)	194 (65.8)		
Marital status								
Married	1078 (53.9)	342 (47.1)	192 (65.1)	< 0.001^a^	337 (49.3)	207 (70.2)	< 0.001^b^	0.143
Unmarried	922 (46.1)	384 (52.9)	103 (34.9)		347 (50.7)	88 (29.8)		
Education level								
College level or higher	1717 (85.9)	594 (81.8)	270 (91.5)	< 0.001^a^	578 (84.5)	275 (93.2)	< 0.001^b^	0.108
Less than college level	283 (14.1)	132 (18.2)	25 (8.5)		106 (15.5)	20 (6.8)		
Employment status								
Employed	1592 (79.6)	508 (70.0)	231 (78.3)	0.007^a^	583 (85.2)	270 (91.5)	0.007^b^	< 0.001^c^
Unemployed	408 (20.4)	218 (30.0)	64 (21.7)		101 (14.8)	25 (8.5)		
Ownership of personal vehicle								
Yes	1445 (72.2)	444 (61.2)	240 (81.4)	< 0.001^a^	498 (72.8)	263 (89.2)	< 0.001^b^	< 0.001^c^
No	555 (27.8)	282 (38.8)	55 (18.6)		186 (27.2)	32 (10.8)		

Abbreviation: SD, Standard deviation.

^a^ Differences among women based on SES.

^b^ Differences among men based on SES.

^c^ Differences based on gender.

### 3.2 Prevalence of the Walking^M^

**FIGURE 1:**
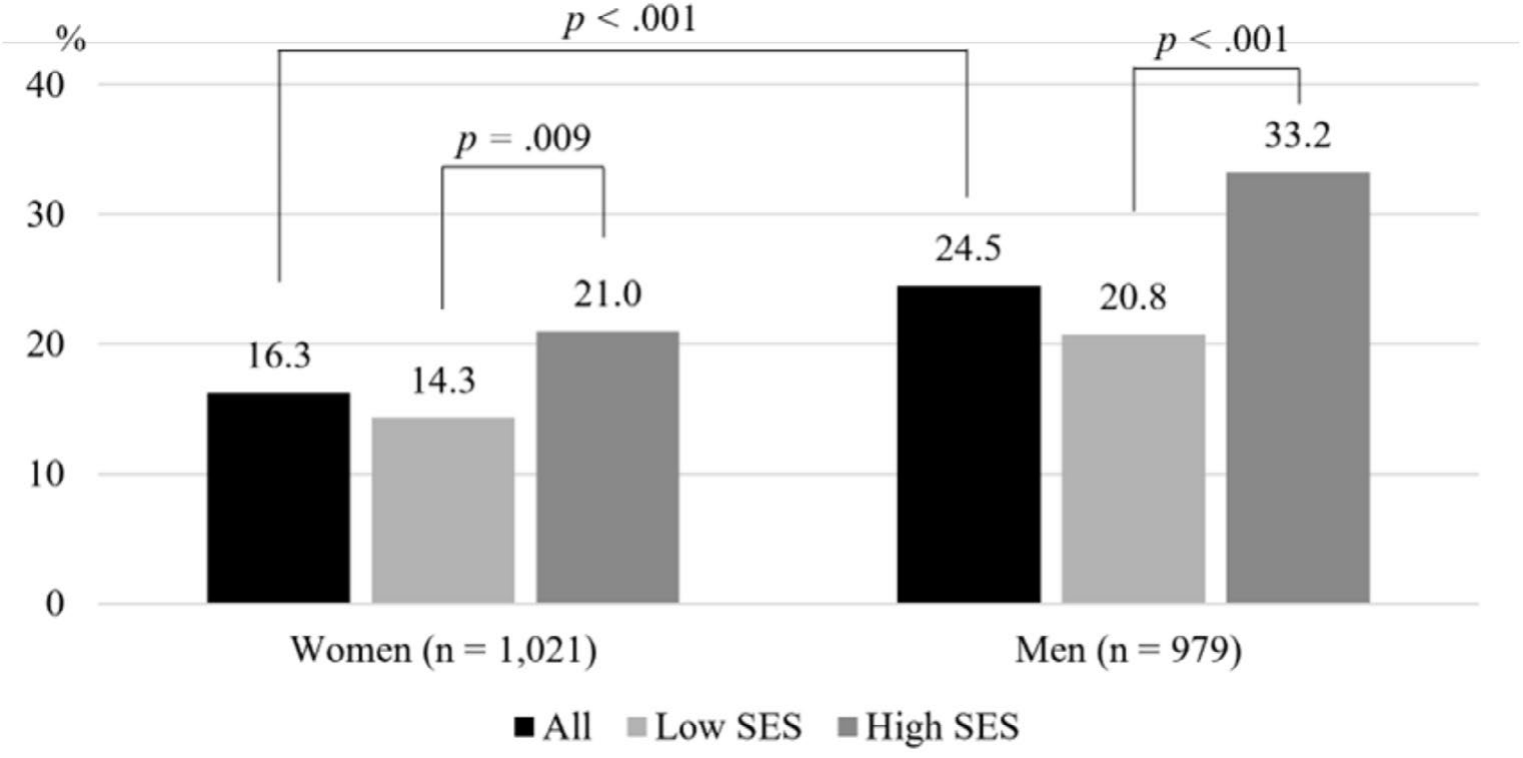
Prevalence of the maintenance of regular walking based on gender and SES. SES, socioeconomic status.

Figure 1 displays the prevalence of walking^M^ by gender and SES. The prevalence was 16.3% among women and 24.5% among men, indicating that men were significantly more likely to maintain regular walking compared to women (*p* < 0.001). Across both genders, individuals in the high SES group exhibited significantly higher adherence to walking^M^ than those in the low SES group (*p* < 0.05). Specifically, 21.0% of women and 33.2% of men in the high SES group engaged in walking^M^.

### 3.3 Perceived Neighbourhood Built Environment Attributes

**FIGURE 2:**
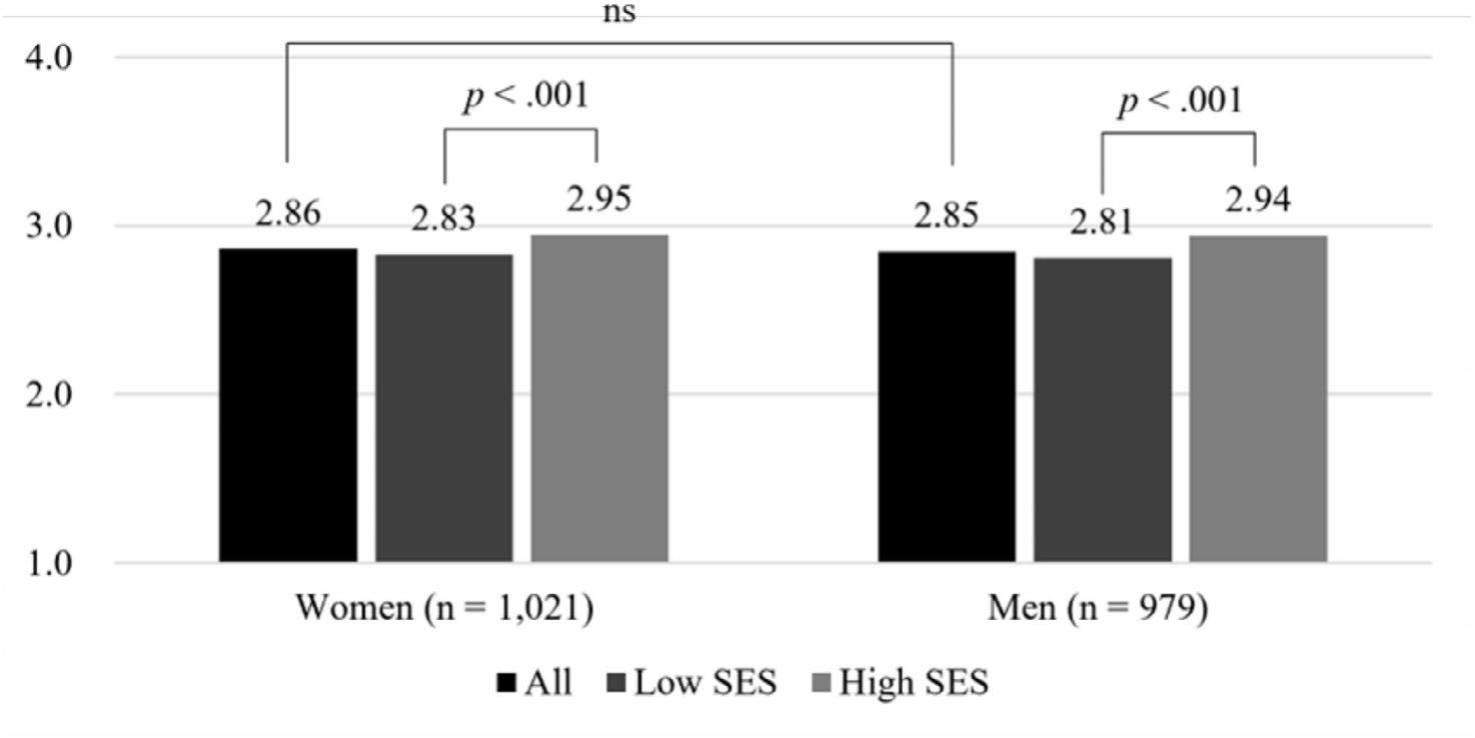
Total score on the PANES based on gender and SES. ns, not significant; PANES, Physical Activity Neighbourhood Environment Scale; SES, socioeconomic status.

Figure 2 illustrates the perceived neighbourhood environment based on the total PANES score, stratified by gender and SES. There was no significant difference in the total score between women and men. However, scores were significantly higher in the high SES group (women: 2.95; men: 2.94) compared to the low SES group (women: 2.83; men: 2.81) across both genders (*p* < 0.001).

Table 2 presents the proportion of individuals who perceived each neighbourhood environmental attribute as walking‐friendly. Across all gender and SES groups, the lowest proportion was observed for perceptions of pedestrian and bicycle infrastructure (31.1%–43.4%). Additionally, fewer than 50% of women and men in the low SES group perceived traffic safety as walking‐friendly. In contrast, more than 90% of participants in all groups perceived residential density and access to public transit as walking‐friendly.

**TABLE 2:** Association between neighbourhood environmental attributes and the maintenance of regular walking (*N* = 2000).

	Women (*n* = 1021)				Men (*n* = 979)			
	Low SES		High SES		Low SES		High SES	
	(*n* = 726)		(*n* = 295)		(*n* = 684)		(*n* = 295)	
	*n* (%)	Adjusted OR	*n* (%)	Adjusted OR	*n* (%)	Adjusted OR	*n* (%)	Adjusted OR
		(95% CI)^a^		(95% CI)^a^		(95% CI)^a^		(95% CI)^a^
Residential density	671 (92.4)	0.8 (0.368–1.562)	279 (94.6)	0.9 (0.272–2.857)	630 (92.1)	1.2 (0.581–2.455)	279 (94.6)	3.5 (0.756–15.952)
Land use mix	538 (74.1)	**2.6 (1.433–4.746)**	227 (76.9)	2.0 (0.927–4.370)	484 (70.8)	**2.3 (1.440–3.710)**	226 (76.6)	**2.0 (1.062–3.886)**
Access to transit	687 (94.6)	1.3 (0.450–3.899)	284 (96.3)	1.3 (0.257–6.307)	635 (92.8)	2.0 (0.814–4.732)	278 (94.2)	1.4 (0.411–4.450)
Aesthetics	401 (55.2)	**1.9 (1.235–3.043)**	215 (72.9)	1.7 (0.810–3.391)	384 (56.1)	**1.9 (1.300–2.872)**	202 (68.5)	1.2 (0.718–2.155)
Pedestrian/Bicycling infrastructure	226 (31.1)	**1.6 (1.028–2.483)**	126 (42.7)	1.8 (0.969–3.214)	229 (33.5)	**1.6 (1.077–2.318)**	128 (43.4)	1.2 (0.707–1.939)
Recreation facilities	510 (70.2)	**2.8 (1.569–4.889)**	236 (80.0)	1.5 (0.658–3.190)	472 (69.0)	**1.9 (1.223–2.974)**	236 (80.0)	1.8 (0.905–3.615)
Street connectivity	433 (59.6)	1.0 (0.644–1.532)	190 (64.4)	1.2 (0.616–2.161)	425 (62.1)	0.9 (0.615–1.320)	189 (64.1)	1.4 (0.843–2.439)
Crime safety	521 (71.8)	1.0 (0.646–1.683)	215 (72.9)	0.9 (0.470–1.824)	496 (72.5)	**2.6 (1.556–4.335)**	225 (76.3)	1.5 (0.815–2.812)
Traffic safety	335 (46.1)	1.0 (0.652–1.524)	132 (44.7)	0.6 (0.346–1.153)	305 (44.6)	1.4 (0.979–2.066)	156 (52.9)	1.2 (0.706–1.919)
Pedestrian safety	541 (74.5)	**2.9 (1.539–5.309)**	247 (83.7)	0.8 (0.355–1.728)	497 (72.7)	1.5 (0.977–2.395)	242 (82.0)	1.7 (0.842–3.475)

*Note:* Bold characters indicate significant findings (i.e., findings with a *p*‐value < 0.05).

Abbreviations: CI, confidence interval; OR, odds ratio.

^a^ Obtained from logistic regression analysis adjusted for age, education level, employment status, marital status, obesity and ownership of personal vehicle.

Among women, those in the low SES group were significantly less likely than their high SES counterparts to perceive aesthetics, pedestrian/bicycle infrastructure, recreational facilities and pedestrian safety as walking‐friendly (*p* < 0.05). Similarly, among men, the low SES group reported significantly lower perceptions of aesthetics, pedestrian/bicycle infrastructure, recreational facilities, traffic safety and pedestrian safety compared to the high SES group (*p* < 0.05).

### 3.4 Association Between Perceived Neighbourhood Built Environment Attributes and Walking^M^

Among women with low SES, the following neighbourhood environmental attributes were significantly associated with walking^M^: land use mix (OR = 2.6; 95% CI: 1.433–4.746), aesthetics (OR = 1.9; 95% CI: 1.235–3.043), pedestrian/bicycling infrastructure (OR = 1.6; 95% CI: 1.028–2.483), recreational facilities (OR = 2.8; 95% CI: 1.569–4.889) and pedestrian safety (OR = 2.9; 95% CI: 1.539–5.309) (Table 2). No significant associations were observed among women with high SES. Among men with low SES, significant associations with walking^M^ were found for land use mix (OR = 2.3; 95% CI: 1.440–3.710), aesthetics (OR = 1.9; 95% CI: 1.300–2.872), pedestrian/bicycling infrastructure (OR = 1.6; 95% CI: 1.077–2.318), recreational facilities (OR = 1.9; 95% CI: 1.223–2.974) and crime safety (OR = 2.6; 95% CI: 1.556–4.335). Among men with high SES, only land use mix was significantly associated with walking^M^ (OR = 2.0; 95% CI: 1.062–3.886).

## 4 Discussion

This study identified notable disparities in the walking^M^ based on gender and SES. Women were significantly less likely than men to maintain regular walking, and individuals with low SES were less likely to do so compared to those with high SES. Neighbourhood built environmental attributes associated with walking^M^ did not differ by gender. Furthermore, participants with low SES were more likely to perceive their neighbourhood environment as less supportive of walking. Importantly, among individuals with low SES, perceived neighbourhood attributes—specifically aesthetics, recreational facilities, pedestrian/bicycling infrastructure, safety and land use mix—were significantly associated with walking^M^.

Our findings indicate that women are less likely than men to sustain walking^M^. This result is consistent with a longitudinal study conducted in Australia, which reported a higher prevalence of walking^M^ among men (Sugiyama, Shibata, et al. 2015). However, the majority of previous studies have suggested that women are more likely than men to engage in leisure walking (Ghani et al. 2016; Pollard and Wagnild 2017), implying they may initiate walking but encounter greater challenges in sustaining it over an extended period, particularly beyond 6 months. Meanwhile, our finding revealed no significant gender differences in the association between walking^M^ and neighbourhood environmental attributes, suggesting that environmental factors alone may not explain gender disparities. Rather, they may stem from a combination of systemic and contextual influences—such as economic constraints, time‐related demands and environmental barriers—that have been identified as critical determinants of walking^M^ among women (Burse et al. 2020; Peng et al. 2023). For instance, women experiencing financial constraints may find it difficult to afford exercise‐related resources such as fitness centre memberships or equipment (Burse et al. 2020). Many women dedicate time to household tasks and childcare during evenings and weekends, which restricts their capacity for the maintenance of physical activity (Burse et al. 2020; Peng et al. 2023). Additionally, the time demands associated with household responsibilities and childcare—especially during evenings and weekends—may limit opportunities for sustained physical activity (Burse et al. 2020; Peng et al. 2023).

Moreover, our findings revealed that individuals with low SES are less likely to sustain walking^M^ compared to those with high SES. As with women, walking^M^ among individuals with low SES appears to be shaped by socioeconomic factors, in addition to environmental factors discussed in the following paragraphs (Caspi et al. 2013; Kamphuis et al. 2009; Koohsari et al. 2017). In particular, economic constraints can limit access to leisure resources and reduce available free time, while the higher prevalence of chronic health conditions in this population may further hinder the ability to maintain consistent walking routines (Kamphuis et al. 2009; Koohsari et al. 2017).

In our study, while gender did not appear to play a significant role in the association between PANES attributes and walking^M^, SES emerged as a key differentiating factor, with notable disparities observed across SES groups. This finding contrasts with prior research on regular walking, which has reported gender‐based differences. For example, studies by Spence et al. (2006) and Liao et al. (2017) identified associations between walking and specific neighbourhood features among women—such as aesthetics, land use mix (e.g., access to shops), presence of sidewalks, availability of recreational facilities and observing others being physically active. In contrast, these studies reported no significant environmental attributes associated with walking among men. These discrepancies suggest that the environmental factors associated with walking^M^ (i.e., sustaining regular walking for 6 months or more) may differ from those associated with general walking behaviour. This highlights the importance of distinguishing between walking initiation and long‐term adherence in the context of environmental influences.

Our study found that SES played a critical role in shaping the relationship between neighbourhood environmental attributes and walking^M^, with individuals of low SES being more strongly influenced by these attributes than their high SES counterparts. While few studies have primarily focused on SES as the primary variable in environmental correlates of walking, prior research has generally addressed regular walking based on weekly duration, not sustained walking^M^. For example, Michael et al. (2010) found that parks and trails influenced walking in high SES older men but not in low SES. In contrast, Sugiyama, Howard, et al. (2015) reported that residential density was positively associated with walking in the low SES group, while walking infrastructure was associated with walking in the high SES group. In our study, conducted in Seoul, the neighbourhood environmental attributes significantly associated with walking^M^ in the low SES group included aesthetics, pedestrian/bicycling infrastructure, recreational facilities and safety. These findings suggested that walking^M^ may be influenced by region‐specific environmental conditions. Furthermore, previous research has shown that individuals in low‐income communities are less likely to have access to sidewalks, adequate street or sidewalk lighting, marked crosswalks and traffic calming measures (Safe Routes Partnerships 2012). Unlike previous studies, our findings indicate that in metropolitan areas like Seoul, aesthetics, infrastructure and safety are particularly important for supporting walking^M^.

Our findings indicate that neighbourhood aesthetics are significantly associated with the walking^M^, particularly among vulnerable populations. This consistently aligns with prior research demonstrating that the presence of visually appealing features in one's neighbourhood encourages walking behaviour (Dadpour et al. 2016; Humpel et al. 2004). Taken together, these findings suggest that beyond merely providing physical spaces for walking, aesthetically pleasing environments may play a critical role in supporting the long‐term walking^M^. Moreover, our findings suggest that the presence of recreational facilities and pedestrian and bicycling infrastructure plays significant roles in supporting the walking^M^ among Seoul citizens, particularly those with low SES. This aligns with Andrade et al.'s study, which further emphasised that access to leisure facilities—shaped by factors such as cost, safety and hygiene—is associated with engagement in leisure‐time physical activity (Andrade et al. 2021). A systematic review by Müller et al. (2024) reported a positive association between pedestrian infrastructure—such as adequate maintenance, sufficient width and the absence of obstructions—and total walking levels.

In this study, safety was identified as a key determinant of walking^M^ among individuals with low SES. Specifically, perceived crime safety—referring to the perceived risk of crime within one's neighbourhood—and perceived pedestrian safety—reflected in the visibility of others engaging in physical activity—were both significantly associated with sustained walking behaviour. Prior research has consistently underscored the role of safety in promoting walking (Aliyas 2020; Chen et al. 2013; Hong and Chen 2014). For instance, Aliyas (2020) reported positive associations between neighbourhood crime and traffic safety and walking engagement, while Hong and Chen (2014) found that individuals living in safe and densely populated areas were more likely to walk regularly. Hong and Chen (2014) demonstrated that traffic and crime safety, along with proximity to service facilities, significantly reduce the risk of insufficient walking. These findings underscore the critical need to enhance both actual and perceived neighbourhood safety as a strategy to support sustained walking, particularly z.

This study has several limitations. First, the cross‐sectional nature of this study limits the ability to draw causal inferences. Future research should employ longitudinal designs to explore how gender‐ and SES‐based differences in walking^M^ evolve over time. Second, as this study relied on self‐reported physical activity data, incorporating objective measures of walking^M^—such as accelerometers or mobile tracking—would enhance the validity and accuracy of future findings. Further research should also consider using Geographic Information Systems (GIS) to more precisely examine the spatial relationship between neighbourhood environmental attributes and walking^M^. Third, because the survey was conducted online, individuals with limited internet access or those unable to use online platforms were excluded. This may have introduced selection bias and affected the representativeness of the study sample, potentially resulting in either over‐ or underestimation of the findings due to digital disparity. In line with the above issue, two socioeconomic indicators—college‐educated individuals (85.9%) and those with employment (79.6%)—are higher than the corresponding national averages in South Korea (Korea Disease Control and Prevention Agency 2021). Therefore, the findings of this study should be carefully interpreted. Finally, as the data were collected in September 2020 during the COVID‐19 pandemic, both walking behaviours and perceptions of the neighbourhood‐built environment may have been influenced, which could have led to an underestimation of the prevalence of walking^M^. Empirically, we found that 20.3% of participants reported walking^M^, which is lower than the 25.8% observed in an Australian longitudinal study (Sugiyama, Shibata, et al. 2015), although the timeframes of the two studies differ considerably. Despite these limitations, this study may offer meaningful insights by uniquely analysing gender‐ and SES‐specific patterns in walking^M^ and their associations with neighbourhood environments in a metropolitan context. The findings inform the development of targeted urban planning and public health strategies for vulnerable populations, particularly women and individuals with low SES. Community health nurses, in particular, emphasise environment‐centred over individual‐level strategies when aiming to improve population health. In this context, the study is significant in that it explores built environmental attributes among health‐disadvantaged groups who are often underrepresented in policy and research.

Building on our findings, policymakers in the Seoul Metropolitan Government should recognise that walking^M^ is a complex, multifaceted issue shaped by a range of environmental factors. Regarding aesthetics, city regulations mandate the allocation of a portion of construction costs to public art installations. In addition, the government continues to enhance the urban landscape and promote walkability through initiatives such as river restoration and landscape improvements (Seoul Solution 2024). In terms of recreational facilities, the Seoul Metropolitan Government has been installing urban parks and trails to provide free access to spaces for physical activity (Seong et al. 2021). Concomitantly, efforts to improve safety include increasing the number of security streetlights to enhance nighttime pedestrian safety (Seoul Solution 2021). However, pedestrian and bicycling infrastructure remain underdeveloped. As of 2022, bicycle roads accounted for only 11% of all roads in Seoul, which falls below the national average of 17% (Seoul Environmental Union 2020a, 2020b). To effectively promote the walking^M^ through environmental enhancements, a comprehensive and integrated approach is essential—one that concurrently addresses neighbourhood aesthetics, access to recreational facilities, safety and the development of pedestrian and bicycling infrastructure in Seoul.

Finally, urban planners, health policymakers and community health nurses may foster healthier and more supportive environments that promote walking^M^, especially for women and low SES individuals. Evidence‐based urban planning and policy initiatives are essential for creating equitable, health‐promoting neighbourhood environments in Seoul.

## 5 Conclusion

Women and the population with low SES are less likely to maintain regular walking compared to men and those with high SES. Neighbourhood environmental attributes—specifically aesthetics, pedestrian and bicycling infrastructure, recreational facilities and safety—were found to be significantly associated with walking^M^ exclusively among the population with low SES, but not among the women population. These findings suggest that targeted interventions aimed at improving these environmental features may enhance the walking^M^, particularly among vulnerable populations. Moreover, the results underscore the importance of comprehensive urban planning and health promotion strategies to reduce health disparities and promote overall community well‐being. In this regard, the study offers insights aligned with environment‐centred approaches emphasised in community health practice, particularly in supporting the health of structurally disadvantaged groups.

## Author Contributions

Jina Choo conceptualised the study, formulated the research question, supervised data collection and curation, supervised data analysis and drafted and edited the manuscript. Songwhi Noh administrated data collection and curation, conducted data analysis and drafted the manuscript. Sooyeon Park contributed to the development of the methodology, administrated the survey and edited the manuscript. Sae Young Jae reviewed the methodology, ensured validation of the study and edited the manuscript. All authors read and approved the final version of the manuscript.

## Disclosure

Statement on number of references: This study investigated the association between 10 neighbourhood environmental attributes and walking maintenance, stratified by gender and socioeconomic status. While only a subset of attributes was discussed in depth, more than 25 references were required to appropriately contextualise the key findings and support their interpretation.

An epidemiologist, Dr. Jina Choo (PhD, DrPH), is part of the author team and reviewed all statistical analyses to ensure methodological accuracy and rigour.

## Ethics Statement

The parent survey did not collect any personally identifiable information. Based on the Enforcement Regulations of the Bioethics and Safety Act, the original survey was conducted without IRB approval (Ministry of Government Legislation 2019). This secondary data analysis was exempted from ethical review by the Institutional Review Board of Korea University (No. KUIRB‐2021‐0035‐02).

## Conflicts of Interest

The authors declare no conflicts of interest.

## Data Availability

The data supporting the findings of this study are available from the corresponding author upon reasonable request.
